# Effect of adjuvant interferon therapy on hepatitis B virus-related hepatocellular carcinoma: a systematic review

**DOI:** 10.1186/s12957-016-0912-7

**Published:** 2016-06-09

**Authors:** Shu Yang, Qi Lin, Wei Lin, Weilei Hu, Guosheng Wang

**Affiliations:** Department of Gastroenterology, No. 202 Hospital of Chinese People’s Liberation Army, No. 5, Guangrong Street, HePing District, Shenyang, Liaoning 110003 China; Department of Pharmacy, Integrated Traditional and Western Medicine Hospital of Taizhou, Taizhou, China; Department of Pediatrics, The First Affiliated Hospital of Guangxi Medical University, Nanning, China; Institute of Translational Medicine, Zhejiang University, Zhejiang, China

## Abstract

**Objective:**

The objective of this study is to evaluate the efficacy of adjuvant interferon therapy for hepatitis B virus-related hepatocellular carcinoma (HCC) after different previous therapy.

**Methods:**

An electronic search for articles about adjuvant treatment with IFN for patients with HCC published between 2000 and 2015 was conducted in MEDLINE, PubMed, Cochrane Library, and EMBASE databases. All data was tested with Stata12.0 software.

**Results:**

Six trials with a total of 1054 subjects were screened according to inclusion and exclusion standards. Five hundred and seventeen HCC patients were treated with adjuvant treatment with IFN and 537 patients with placebo. Compared to the control group, both the recurrence rate and death rate of HCC in IFN group were statistically lower, especially after transhepatic arterial chemotherapy and embolization (TACE) treatment and both TACE and resection according to subgroup analysis.

There is no statistical significance on the both recurrence and death rate of HBV-related hepatocellular carcinoma after surgical resection treatment (RR = 0.96, 95 % CI, 0.84 to 1.1, *p* = 0.59 for recurrence and RR = 0.78, 95 % CI, 0.60 to 1.04, *p* = 0.09 for death rates).

**Conclusions:**

Adjuvant IFN therapy may significantly reduced mortality as well as recurrence rate of patients with HBV-related HCC after no matter what the previous treatment. On the other hand, there is no statistical significance on the recurrence rate and mortality after surgical resection only. More research is needed into the relationship between effect of adjuvant interferon therapy and previous therapy, especially TACE.

## Background

Primary liver cancer is mainly composed of hepatocellular carcinoma (HCC), which is the world’s third most common cause of cancer deaths [1]. About 55 % liver cancer deaths worldwide occur in China, because of the highest age-adjusted incidence of HCC due to chronic hepatitis B (CHB) [2]. Due to the high difficulty in early diagnosis of malignant tumors, most of cancer metastasis have occurred for the first time seeing a doctor [3]. Most patients received surgical resection or transhepatic arterial chemotherapy and embolization (TACE) [4]. Unfortunately, the recurrence rate after 3 years of liver cancer which is treated by pure resection or chemotherapy via hepatic artery embolism is more than 50 %, which is the main cause of death after treatment [5, 6]. Most HCC patients carry with hepatitis virus, which is one of the three main complications of patients with CHB, resulting in one million deaths each year [6, 7] and, of these, nearly 500,000 deaths are in China [2]. Studies have suggested interferon can reduce the recurrence of HCC after treatment, because interferon can inhibit the replication of the hepatitis virus and kill cancer cells, but the conclusion is not consistent [8–11].

In this paper, the clinical data is put forward by evidence-based medicine system evaluation, and a meta-analysis using fixed/random effects model is used to study the influence of recurrence rate and mortality of viral hepatitis liver cancer, which is treated by interferon after surgical resection or TACE or both surgical resection and TACE, and provide the foundation for evidence-based medicine.

## Methods

### Literature retrieval

We reviewed all the experimental results of the curative effect of interferon adjuvant treating patients with HCC. Computerized literature searches of PubMed, the Cochrane Library, and EMBASE database (2005–2015) were undertaken. Search words were “hepatocellular carcinoma,” “liver cancer,” “liver tumor,” “interferon.” Review was conducted with these terms separately in different combinations. In addition, references catalog of relevant original articles and comments were reviewed to find out other qualified trials.

### Inclusion and exclusion criteria

The inclusion criteria are as follows: (1) All the cases were pathologically diagnosed as liver cancer combined with viral-B hepatitis; (2) both interferon group and control group were treated by surgical removal or TACE; (3) the interferon adjuvant therapy was given after the first treatment (surgical removal, TACE) for more than 3 months; (4) the follow-up time must be more than 1 year, and the control group was treated with placebo; and (5) the sample size >35.

The exclusion criteria are as follows: (1) Metastatic liver cancer or recurrent liver cancer has been treated; (2) repetitive articles and small proportion articles; (3) the follow-up time was less than 1 year; and (4) the sample size <35.

### Data extraction

Two reviewers independently extracted all literatures to determine if the relevant trials meet the included criteria.

The study incorporated is reviewed by random method, blinded method, and follow-up distribution plan. According to the Cochrane system evaluation handbook, the creditability of all studies can be divided into three levels (Table 1).

**Table 1:** Quality of assessment of each included studies

Study	Trial type	Random sample	Allocation concealment	Blinded allocation	Lost of follow-up	ITT	Grade
Lo CM	RCT	Adequate	Adequate	Unclear	Yes	Yes	A
Li M	RCT	Adequate	Unclear	Unclear	Yes	Yes	B
Li N	nRCT	Adequate	Adequate	Unclear	Yes	Yes	B
Sun HC	RCT	Adequate	Adequate	Adequate	Yes	Yes	A
Chen LT	RCT	Adequate	Adequate	Adequate	Yes	Yes	A
Chao Zou	nRCT	Adequate	Adequate	Adequate	Yes	Yes	A

### Data analysis

The review management software (Stata) is used for statistical analysis, and all outcomes were expressed as RR with 95 % CI. The Cochrane Q test was used to detect heterogeneity of the effects; significant heterogeneity was defined as a *p* value <0.1. A fixed effects model or random effects model was used depending on the absence or presence of heterogeneity. *I*^2^ statistic was estimated to describe the percentage of the variability attributable to heterogeneity. Studies with an *I*^2^ statistic of <25 % are considered to have no heterogeneity, those with an *I*^2^ statistic of 25–50 % are considered to have low heterogeneity, and those with an *I*^2^ statistic of 50–75 % are considered to have moderate heterogeneity, in which the random effects model was used.

### Retrieval results

Two hundred and nineteen relevant articles were retrieved through combined manual and computerized retrieval in the MEDLINE, PubMed, the Cochrane Library, and EMBASE database from 2005–2015. One hundred and thirty-six articles were excluded, because the title and abstract has nothing to do with the purpose of this meta-analysis. Finally, 6 articles and a total of 1054 subjects were included in the systematic review, including 4 randomized controlled trials and 2 nonrandomized controlled trials.

Four trials examined the effect of IFN after surgical resection and one trial after TACE only and the other after both surgical resection and TACE.

A total of 1054 subjects were included in the final meta-analysis: including 517 HCC patients who were treated with interferon and 537 patients who were treated with placebo. The longest follow-up period was 85.2 months, and the shortest follow-up period was 24.0 months. The maximum sample size was 126, and the minimum sample size was 35. Characteristics of the 6 included studies were listed in Table 2.

**Table 2:** Details of included studies

Author/Ref.	Year/country	Previous therapy	Group	No.	Median age	Gender (M:F)	Interventions	Median follow-up time (m)
Chao Zou	2015	Resection + TACE	IFN	102	53	91:11	IFN	–
			Control	126	52	113:13	IFN + TACE.	
Li N	2010	Resection	IFN	43	53.2	31:12	IFN	–
			Control	36	51.2	26:10	ST	
Sun, HC	2006 China	Resection	IFN	118	52.2	106:12	IFN	36.5
			Control	118	50.4	102:16	ST	
Lo, CM	2007 China	Resection	IFN	40	49	31:9	IFN	Minimum follow-up 30
			Control	40	54	34:6	ST	
Li, MQ	2009 China	TACE	IFN	108	NA	77:31	IFN	24.8
			Control	108	NA	74:34	ST	
Chen, LT	2012 China	Resection	IFN	133	NA	108:25	IFN	63.8 (60.8–66.9)
			Control	135	NA	112:23	ST	

*TACE* transarterial chemoembolization, *ST* symptomatic treatment

### Meta-analysis

**Fig. 1:**
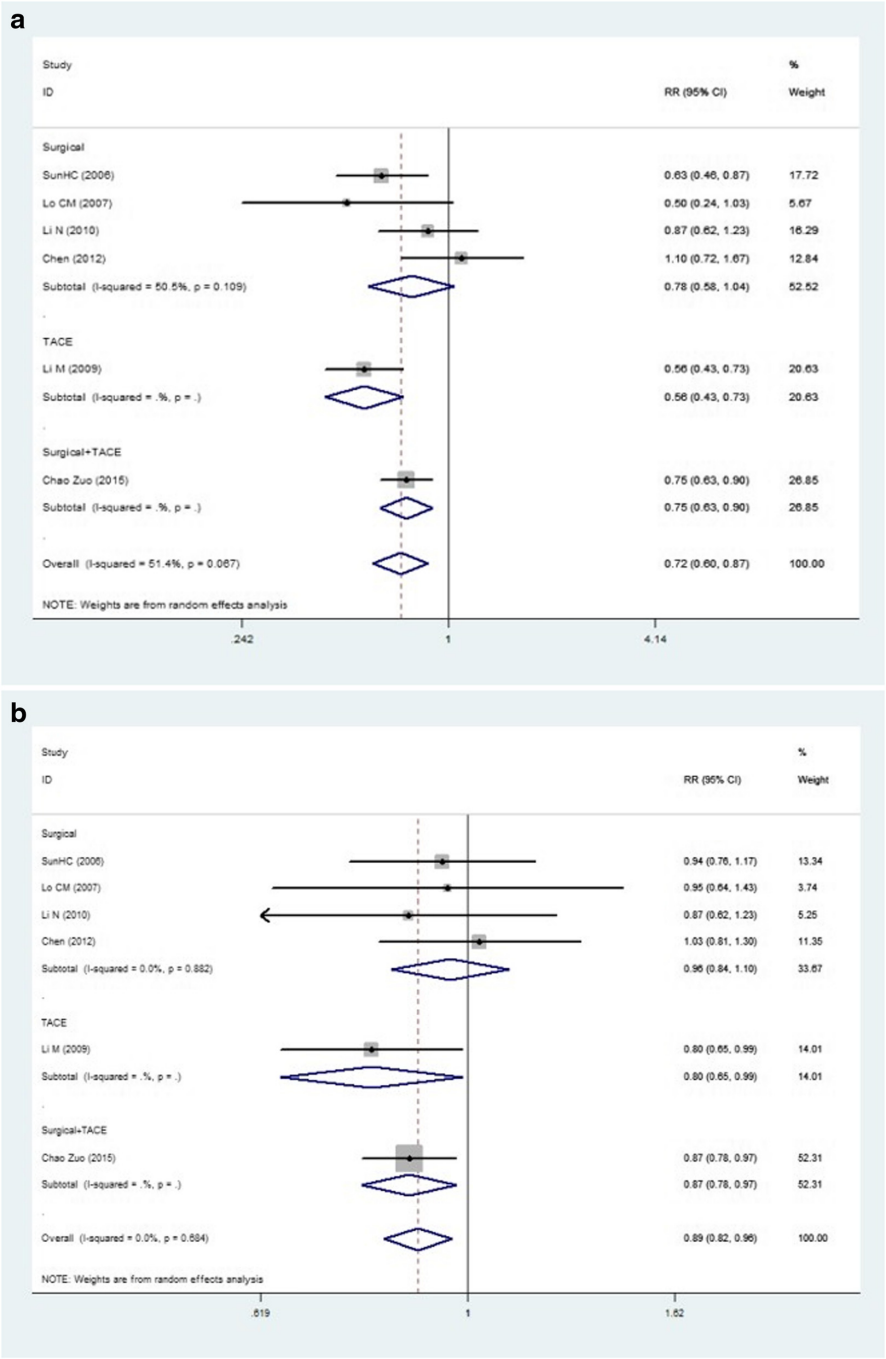
Forest plot of the effect of adjuvant IFN. **a** The comparison between IFN group and control group in the recurrence rates of HCC. **b** The comparison between IFN and control group in the death rates of HCC. *Abbreviation*: *IFN* interferon, *TACE* transarterial chemoembolization. Summary RRs are shown as diamonds, with the middle corresponding to the point estimate and the width representing the 95 % CI

We compare the recurrence rate and mortality between TACE and hepatic resection (HR), both TACE and HR in the treatment of HCC. For further testing differences of the recurrence rate and mortality of different kinds of treatment, we process the subgroup analysis according to the different types of treatment. The meta-analysis results were shown in Fig. 1.

### Recurrence of hepatocellular carcinoma

Six articles [10–15], including 1054 patients, compared the recurrence of hepatocellular carcinoma in the interferon group and the control group. We used “RR” as an indicator of testing method of therapeutic effect and used *X*^2^ test to examine heterogeneity, and the result was *p* = 0.72 (>0.10), *I*^2^ = 0 % (<50 %), which suggested no heterogeneity between the two groups. The fixed effects model was used for meta-analysis. Results (RR = 0.90, 95 % CI, 0.83 to 0.99, *p* = 0.02) showed that interferon adjuvant therapy can significantly reduce the recurrence rate of HCC after initial treatment, especially after TACE treatment and both TACE and surgical resection treatment according to subgroup analysis (RR = 0.80, 95 % CI, 0.65 to 0.99, *p* = 0.04 for TACE; and RR = 0.87, 95 % CI, 0.78 to 0.98, *p* = 0.02 for both TACE and surgical resection). Pooled data analysis revealed that the interferon group had no statistical significance on the recurrence of HBV-related hepatocellular carcinoma after surgical resection treatment (RR = 0.96, 95 % CI, 0.84 to 1.1, *p* = 0.59).

### Death rates for hepatocellular carcinoma

Something similar seems to be happening in death rate. With the same articles, we compared the death rate of hepatocellular carcinoma in the interferon group and the control group. The result was *p* = 0.67 (>0.10), *I*^2^ = 51.4 % (>50 %), and the unfixed effects model was used for meta-analysis. The death rate in the IFN group also significantly decreased according to not only total event analysis (RR = 0.72, 95 % CI, 0.60 to 0.87, *p* = 0.001) but also subgroup analysis (RR = 0.75, 95 % CI, 0.63 to 0.90, *p* = 0.002 for both TACE and surgical resection; and RR = 0.56, 95 % CI, 0.43 to 0.73, *p* = 0.000 for TACE). But, as well, there is no statistical significance on the mortality of HBV-related hepatocellular carcinoma after surgical resection treatment (RR = 0.78, 95 % CI, 0.60 to 1.04, *p* = 0.09).

### Sensitivity analysis and publication bias

**Fig. 2:**
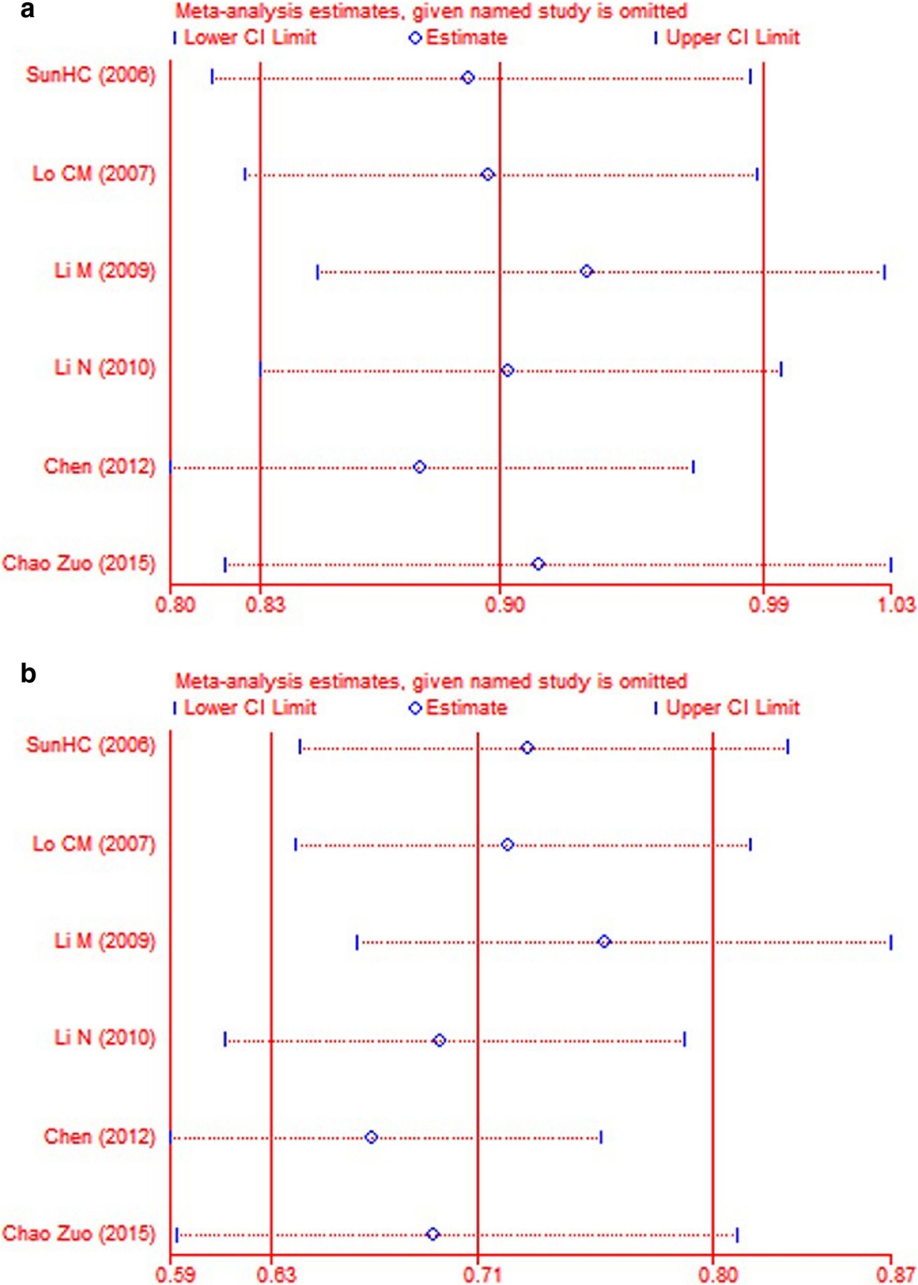
Chart of sensitivity for each comparisons. **a** Recurrence rate of HCC. **b** Death rates of HCC

**Fig. 3:**
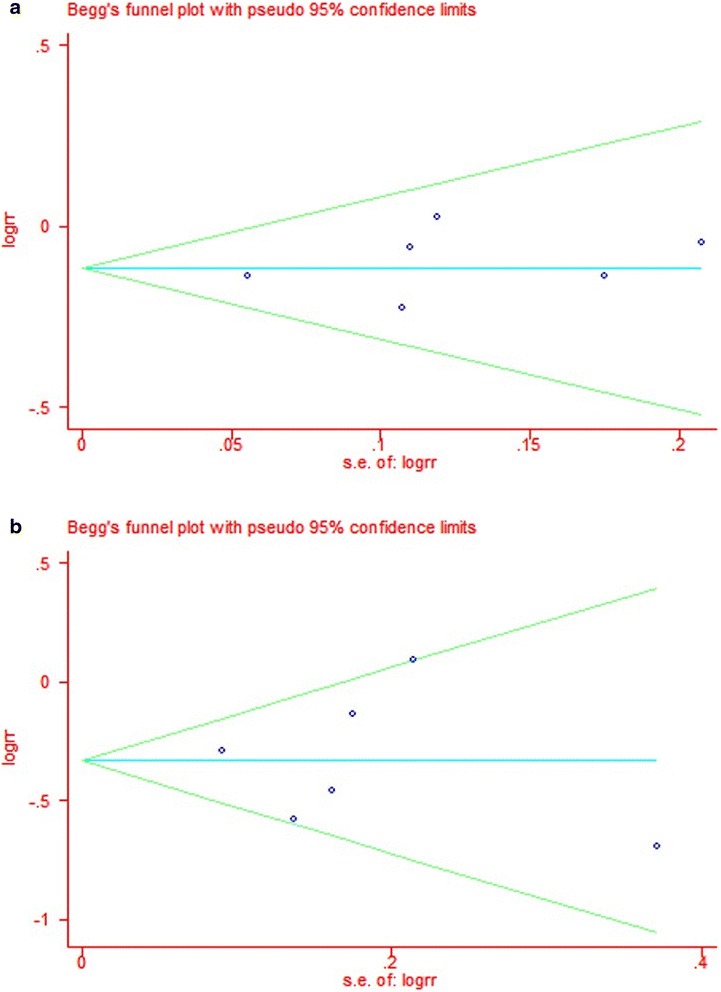
Funnel plot for each comparison. **a** Recurrence rate of HCC. **b** Death rates of HCC

Testing each indicator with fixed/random effects model and visual inspection of chart of sensitivity (Fig. 2), we found that the results are related to each other. We made a funnel chart to compare the including subgroups (Fig. 3). The characteristic of the two funnel charts was basically an inverted funnel and bilateral symmetry, which indicated that there was no publication bias and the conclusion is reliable.

(Begg’s test statistic, *p* = 0.45; Egger’s test statistic, *p* = 0.52 for the funnel plots of recurrence; Begg’s test statistic, *p* = 0.71; Egger’s test statistic, *p* = 0.98 for the funnel plots of death rates.)

## Discussion

HCC is the third leading cause of cancer-related deaths worldwide [1], which has one of the most common risk factors, HBV [16, 17], especially in Asian countries where HBV has a higher incidence [18]. Eighty-five to ninety percent of HCC contain HBV-DNA-integrated tumor cells, providing the strongest evidence for HBV-DNA integration being the primary etiology of CHB-related HCC [19]. Hepatic resection or liver transplantation may provide a complete cure for HCC. However, postoperative recurrence is a big concern [20]. Most recurrences occur within 1 year after surgery. HCC recurrence rate is also relatively high in patients who had liver transplantations [21].

In clinical work, the main method of treatment of liver cancer is surgery and TACE [22]. In order to eliminate deviation caused by different treatment methods, this paper chose the surgery, TACE, and both TACE and surgery resection to treat liver cancer and determined the difference between the two methods through subgroup analysis. The results showed that both the recurrence and mortality of liver cancer had significant differences between interferon group and the control group. But, there is no statistical significance on the recurrence rate and mortality of HBV-related hepatocellular carcinoma after surgical resection only. This result was contrary to the meta-analyses or systematic reviews published before [23]. One of the meta-analysis reported by Zhang included one trial less than ours. And, this trial [15] was published recently by Zuo, and its conclusion was contrary to the trials which were included in Zhang’s report. Meanwhile, samples in Zuo’s report were larger than any other RCTs [10–15], which were all included in our meta-analysis. So, the weight of the trial largely affected the final results, different conclusion achieved is reasonable. Still, it is worth noting that the added article is a nRCT.

Interferon has many biological functions, such as antiviral, antiproliferative, antiangiogenic, and immunomodulatory effects, and it is widely used in the treatment of various diseases [24, 25]. In vitro experiments showed that [26] alpha interferon can inhibit the proliferation of endothelial cells of human umbilical vein, and its inhibition function increased with dose and time increased. In vivo experiments showed that [27] tumor diameter is decreased obviously after adjuvant therapy with interferon, and microvascular density was obviously lower than the control group, and there was significant difference. So, it is presumable that alpha interferon realizes its antitumor effect by inhibiting tumor angiogenesis and anticell proliferation. The above biological characteristics can explain the interferon adjuvant therapy is beneficial for the treatment of liver cancer.

On the other hand, some studies found that the efficacy of IFN on inducing hepatitis Be antigen (HBeAg) seroconversion is not so good [28] and the sustained virologic response rate was only 25 % [29]. But, the main effects of interferon are antiviral, immune regulation, and antiproliferative agents rather than the integration of HBV gene inhibitors [30]. Interferon treatment improves liver fibrosis and liver function by reducing active hepatitis [31], and it can improve overall survival by reducing the severity of the recurrence of the tumor, so it is suitable for secondary therapeutic ablation or resection [32]. From the above description, we can draw the conclusion: interferon does not prevent HBV-related HCC, but there is still a long-term effective effect for viral hepatitis-related HCC.

Several limitations of this study should be considered: (1) Although most of the studies are randomized controlled trials, the sample sizes are relatively small; (2) the basic characteristics of included cases were not identical, and the effect factors (including the clinical stages, pathological pattern, therapeutic method, the size of the tumor, and the body’s immune ability), which influenced clinical prognosis of hepatocellular carcinoma, may affect the results of the meta-analysis; and (3) the included studies mostly reported the recurrence rate and mortality, but there are less reports on overall survival rate and disease-free survival (DFS) of interferon adjuvant therapy.

## Conclusions

In short, the meta-analysis, which included six literatures, indicated that interferon adjuvant therapy can significantly reduce the recurrence rate of viral hepatitis-related hepatocellular carcinoma and improve the survival rate of patients after no matter previous treatment is given including surgical resection and TACE. On the other hand, there is no statistical significance on the recurrence rate and mortality of HBV-related hepatocellular carcinoma after surgical resection only. More research is needed into the relationship between effect of adjuvant interferon therapy and previous therapy, especially TACE.
